# Study on Dicarboxylic Acids in Aerosol Samples with Capillary Electrophoresis

**DOI:** 10.1155/2014/498168

**Published:** 2014-03-09

**Authors:** Heidi Adler, Heli Sirén

**Affiliations:** ^1^Neste Oil Corporation, Technology Center Kilpilahti, P.O. Box 310, 06101 Porvoo, Finland; ^2^Department of Chemistry, University of Helsinki, A.I.Virtasen aukio 1, P.O. Box 55, 00014 Helsinki, Finland

## Abstract

The research was performed to study the simultaneous detection of a homologous series of **α**, **ω**-dicarboxylic acids (C_2_–C_10_), oxalic, malonic, succinic, glutaric, adipic, pimelic, suberic, azelaic, and sebacic acids, with capillary electrophoresis using indirect UV detection. Good separation efficiency in 2,6-pyridinedicarboxylic acid as background electrolyte modified with myristyl trimethyl ammonium bromide was obtained. The dicarboxylic acids were ionised and separated within five minutes. For the study, authentic samples were collected onto dry cellulose membrane filters of a cascade impactor (12 stages) from outdoor spring aerosols in an urban area. Hot water and ultrasonication extraction methods were used to isolate the acids from membrane filters. Due to the low concentrations of acids in the aerosols, the extracts were concentrated with solid-phase extraction (SPE) before determination. The enrichment of the carboxylic acids was between 86 and 134% with sample pretreatment followed by 100-time increase by preparation of the sample to 50 **μ**L. Inaccuracy was optimised for all the sample processing steps. The aerosols contained dicarboxylic acids C_2_–C_10_. Then, mostly they contained C_2_, C_5_, and C_10_. Only one sample contained succinic acid. In the study, the concentrations of the acids in aerosols were lower than 10 ng/m^3^.

## 1. Introduction

Aerosol particles affect human health and participate in climate interaction, acid precipitation, and visibility reduction on the globe. The study of organic aerosols and their chemical composition is essential in understanding many processes in the atmosphere, such as solar radiation scattering, cloud condensation as nuclei actions in cloud formation, and participation in oxidation processes [1, 2].

Individual monocarboxylic acids and *α*, *ω*-dicarboxylic acids (DCAs), as well as *n*-alkanoic acids, *n*-alkenoic acids, and aliphatic DCAs, have been identified as the major organic compounds in aerosols and coniferous forests [3, 4]. Especially aerosol samples from urban, continental background and remote marine sites have been of interest. It has been shown that a majority of aerosols consist of organic compounds containing hydroxyl groups from alcohols and/or carboxylic acids [5–11]. It has also been proven that the possible sources of carboxylic acids (CA) are anthropogenic and biogenic emissions and photochemical reactions, particularly between olefins and ozone [12–17]. In many studies, oxalates have been observed as the most abundant organic species in atmospheric aerosols, and therefore, they have traditionally been studied by many research groups [13, 18]. In spite of that, the research interest has also focused on longer chained DCAs due to their stability against analytical procedures in sampling and separation. Yang et al. noticed in their physical and chemical studies on C_2_–C_9_ DCAs that succinic acid (C_4_) exhibits the lowest photooxidation rate in gas, while oxalic acid has the highest rate in a liquid-phase reaction system [17]. Oxalic acid (C_2_) is the end product of the oxidative decomposition of various organic precursors. Generally, the predominance of oxalic acid (C_2_) is up to 50% of the total atmospheric DCAs, but also malonic (C_3_) and succinic (C_4_) acids have been observed in aerosols from distinctly different sites. [19–25] Clegg et al. [19] have reported that oxalic acid (C_2_) in the atmosphere is partitioned almost completely into aqueous aerosol droplets, except under conditions of low relative humidity, low aerosol pH, and temperatures greater than +15°C. This is understandable since the acids are easily ionised and water soluble. At present, it is well known that atmospheric aerosols contain not only salts and nonvolatile compounds but also volatile and semivolatile organic compounds [6, 9].

Thermodynamic properties of DCAs have been studied in aerosols by Koponen et al. [24]. The work provided thermodynamic data—in particular, liquid-state saturation vapour pressures of three common slightly water-soluble secondary organic aerosol components, namely, malonic (C_3_), succinic (C_4_), and glutaric (C_5_) acids. Longer chain DCAs (adipic (C_6_), pimelic (C_7_), suberic (C_8_), and azelaic (C_9_) acids) have been determined previously by Bilde [26].

To identify the organic compound aerosols, selective and sensitive separation for the sample compounds is needed. Various in-line couplings of separation, identification, and sample preparation devices have been modified for the purpose of concentrating volatile acids [6–9, 25]. Extended sample pretreatment and quite long analysis times dominate the use of chromatographic methods [27–29]. Traditionally, analyses of CAs have been conducted by using gas chromatography (GC) [30–32].

Our group has earlier published the use of capillary electrophoresis (CE) determination of CAs in various matrices [8, 28]. But, there are also other research groups who have extensive experience in CE methodologies for DCAs in various matrices, for example, in food products [33–35]. Due to the many possibilities of separation and detection in CE, the technique is favourable for simultaneous analyses of various groups of compounds, like CAs and DCAs. In addition, the technique is capable to separate CAs from inorganic anions and cations [28, 36].

We have also earlier developed and optimised a CE electrolyte solution for DCAs separation [8]. Then, three different chromophores (2,3-pyrazinedicarboxylic acid, 2,6-pyridinedicarboxylic acid, and 2,3-pyridinecarboxylic acid) were tested and the most suitable of them was chosen for the further studies. It contained 2,6-pyridinedicarboxylic acid as the chromophore for detection and myristyl trimethylammonium bromide as the surfactant to make the separation fast. At that time, one application of analysis of aerosol sample was tested. Qualitative screening showed malonic, succinic, adipic, and suberic acids. The samples were detected in the water-soluble fraction of the aerosol, which was obtained by a commercial microfibre filter. Analytes were extracted from the filter with water in an ultrasonicator. DCAs were identified with reference chemicals, which were spiked into the samples.

In this project, we have studied pretreatment, separation, and concentration techniques that can be online coupled as preseparation techniques with capillary electrophoresis. The studies were performed with anions of homologous series of dicarboxylic acids C_2_–C_10_ (oxalic (C_2_), malonic (C_3_), succinic (C_4_), glutaric (C_5_), adipic (C_6_), pimelic (C_7_), suberic (C_8_), azelaic (C_9_), and sebacic (C_10_) acids). The novelty of the study is the obtained knowledge about the suitability of sample pretreatment techniques to enrich DCAs in environmental aerosol samples. The effectiveness of membrane extraction and solid-phase extraction was also compared.

## 2. Experimental

### 2.1. Chemicals

Chemicals for the electrolyte solutions were 2,6-pyridinedicarboxylic acid (2,6-PDC) (Sigma-Aldrich, Steinheim, Germany), myristyltrimethylammonium hydroxide (MTAH) (Waters, Milford, USA), and methanol (Rathburn, Walkerburn, Scotland). Oxalic acid (C_2_O_4_H_2_, purity 99%, p*K*_*a*1_1.27, p*K*_*a*2_ 4.27), malonic acid (C_3_H_4_O_4_, purity 99%, p*K*_*a*1_ = 2.83, p*K*_*a*2_ = 5.69), succinic acid (C_4_H_6_O_4_, purity 99%, p*K*_*a*1_ = 4.2, p*K*_*a*2_ = 5.6), glutaric acid (C_5_H_8_O_4_, purity 99%, p*K*_*a*1_ = 4.32, p*K*_*a*2_ = 5.54), adipic acid (C_6_H_10_O_4_, purity 99%, p*K*_*a*1_ 4.43, p*K*_*a*2_ 5.41), pimelic acid (C_7_H_12_O_4_, purity 99%, p*K*_*a*1_ 4.46, p*K*_*a*2_ 5.58), suberic acid (C_8_H_14_O_4_, purity 99%, p*K*_*a*1_ 4.526, p*K*_*a*2_ 5.498), azelaic acid (C_9_H_16_O_4_, purity 99%, p*K*_*a*1_ 4.550, p*K*_*a*2_ 5.598), and sebacic acid (C_10_H_18_O_4_, purity 99%, p*K*_*a*1_ 4.720, p*K*_*a*2_ 5.450) were from Sigma-Aldrich (Sigma-Aldrich Finland, Helsinki, Finland). Mandelic acid (MA, separation standard, IS_1_) and trichloroacetic acid (TCA, extraction standard, IS_2_), tri-*n*-octylphosphine oxide (TOPO), dissolved in dihexyl ether (DHE), and tris[hydroxymethyl]-aminomethane (TRIS, p*K*_*a*_ = 8.06) were from Fluka (Buchs, Switzerland) and Merck (Darmstadt, Germany). A 0.1 M solution of sodium hydroxide was prepared from NaOH pellets (Akzo Nobel, Bohus, Sweden). Distilled water was deionised (Milli-Q instrument, 18 MΩ cm^−1^ at 25°C) before use. All chemicals were of reagent grade.

### 2.2. Preparation of Standards and Solutions

Stock solutions of 1000 mg/L were separately prepared from nine DCAs. Concentration calibration was performed by diluting the C_2_–C_10_ stock solutions and making them as DCAs standards. The obtained data was used for calculation of calibration with five concentration levels (1 mg/L, 5 mg/L, 10 mg/L, 20 mg/L, 50 mg/L, and 100 mg/L) for each of the DCAs. The results are listed in Table 1.

The final analyses were done in an optimised electrolyte (BGE) containing 4 mM 2,6-PDA and 0.5 mM MTAH at pH 11.0. Before use, the electrolyte solution was filtered through Gelman Acrodisc CR PTFE syringe filters (13 CR, 0.45 *μ*m, Gelman Sciences, Ann Arbor, MI, USA). The suitability of the chemical and instrumental parameters was cross-checked with standards concentration at 5 mg/L.

### 2.3. Sampling and Sample Preparation

The investigated samples in the study were taken on a roof of a high-rise building (four floors) in the Kumpula Campus area (5 km from the centre of Helsinki, Finland). The samples were immobilised from aerosols onto cellulose membrane ultrafilters (pore size 0.2 *μ*m, PM_2.5_) by using a cascade impactor (12 stages). The filters were put in series inside the device. A 24-hour sample collection was used at a 90 m^3^/h flow rate. For extraction one 25 × 25 mm^2^piece of the PM_2.5_ membrane was cut from the filter with a special cutter. For one determination three parallel sample lots were used.

The filter pieces were diluted into 5 mL of Milli-Q water during 60 minutes. The extract was purified with solid-phase extraction (Oasis HLB) using methanol as the eluent. The solvent after addition of a few *μ*L of 0.1 M NaOH was evaporated under a nitrogen atmosphere, followed by dissolving precipitation in either Milli-Q water (200 *μ*L).

The cascade impactor was used for collection of the aerosol samples for the research. In addition to the cascade impactor collection, aerosols were also sampled on 47 mm Whatman QM-A quartz microfibre filters (Whatman International Ltd., Maidstone, UK). The sample collection was made at a flow rate of 20.5 mL/min for 48 h. Prior to use, the filters were preheated at +500°C for 10 h to remove organic contaminants. Analytes were extracted from the whole filter with 5 mL of Milli-Q water with ultrasonic agitation for 20 minutes. The extracts were stored at −20°C until the analyses started. They were used for optimisation of sample pretreatment (ultrasonic agitation, hot water extraction, membrane extraction, and solid-phase extraction). Concentration of DCAs in their mixture in the optimization studies was 5 mg/L.

### 2.4. Extraction

#### 2.4.1. Extraction with Ultrasonic Agitation

Ultrasound extraction was used for the isolation of the DCAs retained on the filters during the sampling of urban aerosols in the environment. The extraction was optimised with real samples, which were spiked with trichloroacetic acid (TCA) that was used as the extraction standard and the internal standard (IS_2_). Due to the simplicity of the instrument used in the procedure, only the extraction time could be optimised (between 30 and 60 minutes). Finally, the extraction time of 60 minutes was chosen due to the 100% enrichment of the external and internal standards, mandelic acid (MA, IS_1_) and TCA (IS_2_).

#### 2.4.2. Hot Water Extraction

Hot water extraction (HWE) was tested as a comparison method to the ultrasonic agitation. Earlier, it has been shown that HWE is an excellent choice for sample processing of aromatic compounds, pesticides, and bromated retardant compounds [7]. The extractions were performed with the collected aerosol samples containing the internal standard TCA (IS_2_). The extraction time was increased with optimisation testing from 30 to 75 minutes. The temperature during extraction was 60°C, being low enough to keep water vapour consumption as minimal as possible [37]. Therefore, grinded caps were used on the tops of the glass tubes.

#### 2.4.3. Solid-Phase Extraction

The samples were extracted with a Supelco SPE instrument (Sigma-Aldrich Finland, Helsinki, Finland). The solid-phase materials (SPE) were Oasis HLB (polymeric reversed-phase polymeric sorbent, Waters Finland, Helsinki, Finland), Strata X (reversed-phase functionalised polymeric sorbent, Phenomenex, Copenhagen, Denmark), and Isolute 101 (strong nonpolar (hydrophobic) polystyrene-divinylbenzene copolymer sorbent) and IsoluteSAX (quaternary amine functionalised silica based sorbent) (IST, Hengoed, UK). The amine functionalised sorbent was JTBaker Amino (J.T.Baker, Deventer, Holland) adsorbent. It was an ion-exchange chromatography sorbent. The steps in the SPE extraction were as follows: conditioning with methanol and water, 1 mL each, sample loading of 5 mL (carboxylic acid standard mixture, 5 mg/L), washing with a methanol-water solution (5 : 95, v/v), and eluting with 1 mL of methanol. After completing the elution, the eluate was evaporated under nitrogen, and the precipitate was diluted into 50 *μ*L of Milli-Q water. The SPE treatment of the samples was made with three replicates and each of them were analysed with six repetitions with CE-UV.

#### 2.4.4. Membrane Extraction

The membrane extraction device was self-constructed in the laboratory following the instructions published by Lüthje [37]. The materials in the support were a porous PTFE membrane (TE 35 Membrane filter, Schleicher GmbH, Dassel, Germany) in between the acceptor and donor devices, with a solution of 10% TOPO (tri-*n*-octylphosphine oxide) dissolved in DHE (dihexyl ether) as an impregnator of the membrane and TRIS (tris[hydroxymethyl]-aminomethane) as an acceptor (AC), and Milli-Q water producing the flush in the donor side. The acceptor (AC) and donor (DC) chemicals were prepared in Milli-Q water.

#### 2.4.5. ME Instrument Optimisation

The ME instrument [38, 39] was optimised by using the AC made of TRIS (pH 7.5) and with a DCA standard mixture solution made into acidified water (5 mL, pH 2.0, adjusted with HCl). The flow rate in the sample recycling before extraction was 0.15 mL/min for 45 minutes (giving 1.5 times the original sample volume). Between the sample pretreatments, the membrane system was washed with water and the TRIS solution for 15 minutes at a flow rate of 0.2 mL/min.

#### 2.4.6. Optimisation of Sample Access and Collection

In membrane extraction the flow rate of the sample was 0.15 mL/min. The whole air sample made to 5 mL was recycled in the ME system once, twice, and thrice within 33, 67, and 100 minutes, respectively. Each cycled fluid was analysed as one sample. Internal standard TCA (25 *μ*L of the IS_2_ standard at pH 2 water) was used in all experiments. Repetitions of the air samples were performed as planed in the measurement design. The best quantitative results were obtained with 67 min recycling time.

#### 2.4.7. Optimisation of the pH in the Acceptor Solution

The sample was twice recycled (flow rate 0.15 mL/min, running time 67 minutes) before analyses. The effect of the pH was studied by buffering the sample to pH 7.0, 8.0, and 9.0. Between the analyses, samples to measure memory effects were taken, after washing for 15 minutes at the flow rate of 0.2 mL/min with water-TRIS solvents (donor-acceptor). Calibration was performed with a mixture containing 25 *μ*L of TCA (stock 1000 mg/L) in a 5 mg/L DCAs mixture. The obtained concentrations for 2.5, 5, 10, 20, 50, 100, 200, and 400 mg/L TCA (optimised as the volume of ME permeate) were 2.991, 4.975, 9.901, 19.61, 47.62, 90.91, 166.67, and 285.7 mg/L, respectively.

The enrichment factor (EF) for the DCAs was calculated according to the following equation:

(1) $$\begin{array}{c} \text{EF}=\frac{C_{a}\;\;\left(\text{final}\right)}{C_{s}\;\;\left(\text{initial}\right)}, \end{array}$$

where *C*_*a*_ (final) is the concentration of the enriched sample injected into CE, and *C*_*s*_ (initial) is the concentration of the DCA in the ultrasonicated sample.

### 2.5. Instruments

A Hewlett-Packard CE System (Agilent Technologies, Waldbronn, Germany) equipped with a photodiode array UV detector was used for the analyses. The compounds were detected with an indirect UV mode using wavelengths of 310 nm as the pilot signal and that of 266 nm as the reference signal. The wavelengths were optimised using oxalic (C_2_) and sebacic (C_10_) acids. Injection was carried out at 50 mbar for 6 sec. The separation voltage was −24 kV (reversed polarity) and the migration window for the carboxylic acids was set to five minutes. The fused-silica capillaries (Composite Metal Services, The Chase, UK) had dimensions of 58.5 cm in length (effective length 50 cm), 50 *μ*m I.D., and 375 *μ*m O.D. The temperature during the analyses was maintained at +25°C with ventilation. The sample tray was also kept at 25°C with a water cooling system.

The new capillaries were conditioned by flushing at high pressure (150 kPa) with 0.1 M NaOH, water and the electrolyte solution for 20 min, 15 min, and 20 min, respectively. Between analyses, the capillary was flushed with the electrolyte solution for 3 min.

A Meter Lab PHM 220 laboratory pH meter (Radiometer, Copenhagen, Denmark) was used for the pH measurements. The combination electrode was calibrated with standard solutions of pH 4.00, 7.00, and 10.00 manufactured by Merck (Darmstadt, Germany). The distilled water was further purified with a Milli-Q apparatus (Millipore, Molsheim France).

## 3. Results and Discussion

### 3.1. Optimization of Separation

**Figure 1:**
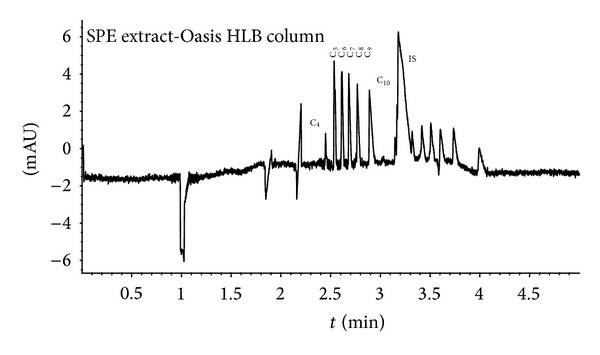
Electropherogram of standard after SPE. The standard mixture contained C_4_–C_10_, TCA, and nonidentified long-chain dicarboxylic acids (C_12_–C_18_). The analyses were performed with two replicates and six repetitions. Experimental conditions: 4 mM PDA + 0.5 mM MTAH, pH 11.0; indirect detection at 310/266 nm; sample injection for 6 sec at 50 mbar; separation voltage −24 kV; analysis time 5 min. DCAs marked in the figure as C_4_–C_10_. IS is the internal standard. After IS migrating peaks are C_14_–C_18_ DCAs. They were not included in the main study. Other experimental details in Experimental.

The studied CDA anions of oxalic, malonic, succinic, glutaric, adipic, pimelic, suberic, azelaic, and sebacic acids were separated with capillary electrophoresis (CE) with indirect UV detection in an optimised electrolyte solution made of 4 mM 2,6-PDA and 0.5 mM MTAH at pH 11.0. The migration order of the DCAs was correlated with their molecular size and the first ionisation constant (p*K*_*a*1_) of the acids (Figure 1). Determination of DCAs was carried out with CE due to its good separation efficiency in 2,6-pyridinedicarboxylic acid electrolyte. Myristyl trimethylammonium bromide was changed to its hydroxide salt, which was needed to reverse the electroosmosis and to get faster analysis get faster analysis and to enhance sensitivity in UV detection. Due to the development, sebacic acid could be studied at concentration range similar to the other DCAs.

TCA and MA were the best candidates for the internal standards (IS_1_ and IS_2_) to find out the operational changes of the overall concept and the method in the separation of the C_2_–C_10_ DCAs. In spite of that, MA was only used for evaluation of the experimental parameters, because it migrated too early and overlapped partly with both monocarboxylic acids (C_1_ and C_2_) and inorganic anions (sulphate and chloride), which had migration times (*t*_*M*_) between 3.6 and 3.9 min, when real samples were studied. Furthermore, it was sensitive to the ionic strength changes in the sample in the tasks from extraction to separation. The identification of the DCAs in the samples was done by spiking the aerosol extracts with CDA standards made to 1–5 mg/L solutions. The final method was used to optimise sample pretreatment and concentration of some aerosol samples. Furthermore, they were used to quantify the aerosol samples which were collected with micropore filters and membrane ultrafilters and microfiber filters.

### 3.2. Sample Extraction

Aerosol particles with DCAs were extracted from cellulose ultra filters of cascade impactor, which were used for quantification and from Whatman QM-A quarz microfibre filters. Microfibre filters were used for method development and choosing the best sample pretreatment technique between utrasonic agitation (US) and hot water extraction (HWE). The performance of US and HWE were compared in extraction of DCAs from the filters into purified water. The result showed that ultrasonic agitation gave better recoveries and it was fast; the extractions were made with US, which is the most usable due to high recoveries of TCA, MA, and DCAs obtained in 60-minute treatment.

### 3.3. DCA Enrichment

**Figure 2:**
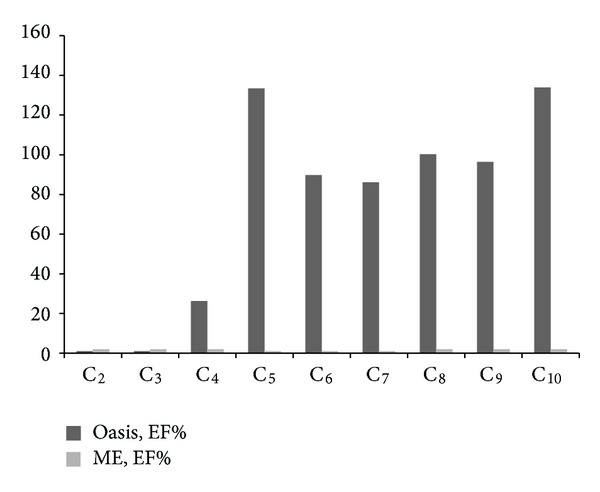
Comparison of enrichment in ME after circulating the samples twice with those made with SPE (Oasis HLB). Sample 25 × 25 cm^2^. The order of the analytes C_1_–C_10_ is from left to right. The DCA concentration is 5 mg/L. The results are averages of six replicates. The calculations are based on electropherograms of the standard mixture that were analysed after sample collection from the acceptor solution. 100% is normal recycling (see Experimental) and 2x recycling is twice the same sample volume (limited with timing). Analysis conditions as described in Experimental.

**Figure 3:**
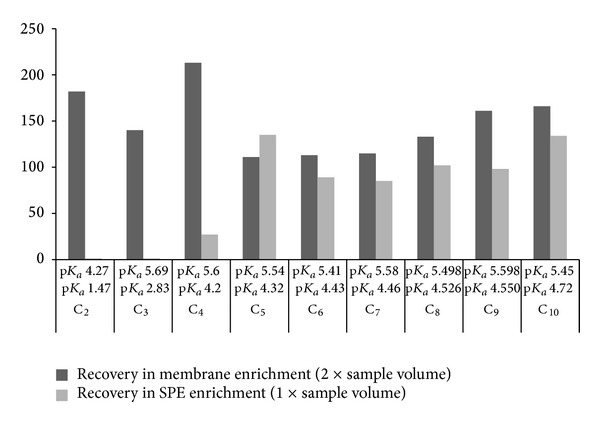
Comparison of enrichment in ME after circulating the samples twice with those made with SPE (Oasis HLB). Sample 25 × 25 cm^2^. The order of the analytes C_1_–C_10_ is from left to right. The DCA concentration is 5 mg/L. The results are averages of six replicates. The calculations are based on electropherograms of the standard mixture that were analysed after sample collection from the acceptor solution. 100% is normal recycling (see Experimental) and 2x recycling is twice the same sample volume (limited with timing). Analysis conditions as described in Experimental.

DCAs concentrations are low in the US extracted aerosol samples. Therefore enrichment prior to CE separation was needed to increase their detectability for indirect UV identification. Thus, we chose solid-phase extraction (SPE) and membrane enrichment (ME) for their concentration tests. Because only one of the methods was planned to be used, the performance and superiority of them for the aerosol cases were studied. First, the ME method with the reagents and the membrane was modified and further optimized based on the studies published in literature [38, 39]. The optimisation was focused on acceptor buffer concentration and its pH, the flushing and collection timings of donor and acceptor solvents, the solvent flow rate during extraction, and the circulation time of the donor solution. The results show (Figure 2) that PTFE membrane worked selectively by improving the sensitivities of IS_2_ and C_2_–C_10_ by 726% and between 15 and 25%, respectively. ME with the chosen parameters gave the highest enrichment for C_2_–C_4_ and medium extractability for C_8_–C_10_. It was observed that acids with low p*K*_*a*1_ could be moved to acceptor solution better than the DCAs with high p*K*_*a*_ values (Figure 3). The longer chain acids did not have high diffusion through the membrane, although the acceptor solution tris[hydroxymethyl]-aminomethane (TRIS, p*K*_*a*_ = 7.5) was ionised to aid the extraction of the anions. It was noticed that the lower ionic strengths in the acceptor solvents were more efficient than the stronger solutions. The ME extraction efficiency and memory effects were also investigated in more detail.

The results showed that optimal ME concentration for the organic acid anions could be obtained, when the sample was two times recycled at optimal donor solution of TRIS (amine) at pH 7.5. However, there were still after the procedure memory effect from the sample at 0.15 mL/min flow rate. It was four to seven times more efficient for C_4_–C_10_ DCAs than the electrolyte solutions modified to pH 8.0 and 8.5.

Low flow rates should be preferred for repeatable and quantitative separation recoveries in ME. As expected, recycling of the sample (study carried out with one to seven cycles) improved the recoveries. However, for the DCAs concentrations in one cycle were only improved with 15%–25% from that of pure solvent extraction reported earlier [40]. To improve the sensitivities, 67 min recycling time would be needed. However, the time was too long. One disadvantage was the memory effect of the DCAs in the ME. Our studies resulted in conclusion that the membrane would need more than 2-hour water cleaning before a new aerosol sample would be introduced to prevent contamination.

### 3.4. Solid-Phase Extraction

**Figure 4:**
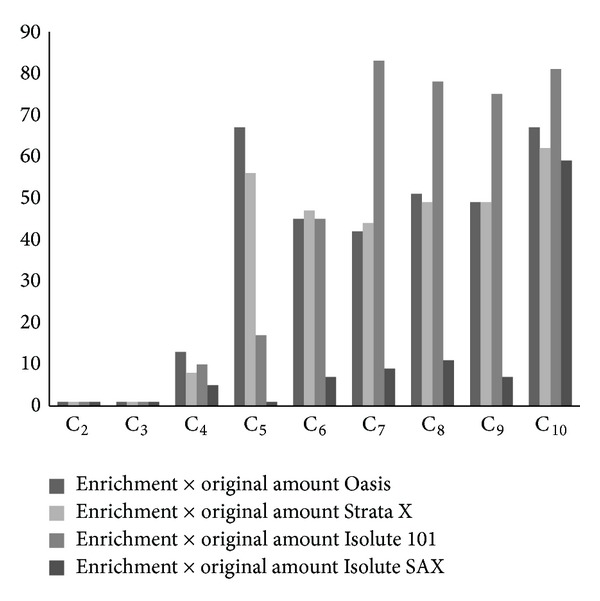
Comparison of the performance enrichment of SPE materials in extraction of the C_2_–C_10_ DCAs. The DCA concentration is 5 mg/L. The results are averages of six replicates. Analysis conditions as described in Experimental and in Figure 1.

Solid-phase extraction (SPE) was preferred over membrane extraction (ME). SPE was used after solvent extraction of the DCAs after extraction from the impact filters. SPE materials (Oasis HLB, Strata X, Isolute 101 and SAX, and J.T.Baker NH_2_ and Quaternary Amine) were tested for the offline concentration and purification of the air samples. The recoveries of the DCAs are shown (Figure 4) with four SPE materials, excluding the Baker materials that could not be optimized for the aerosol samples. Oasis HLB and Strata X were the best for further looking in terms of low inorganic matrix background and good extraction efficiency followed by intensive response for anions of oxalic (C_2_), malonic (C_3_), succinic (C_4_), glutaric (C_5_), adipic (C_6_), pimelic (C_7_), suberic (C_8_), azelaic (C_9_), and sebacic (C_10_) acids. Oasis HLB and Strata X enhanced selectivity for nonspecific analytes with nonpolar retention mechanism on the material surface that was either end-capped or non-end-capped polymer. As seen in Figure 4, Isolute 101 sorbent was even superior to Oasis and Strata X materials, when the acids C_7_–C_10_ were studied, but it was not used for the further studies due to its 50% higher capability to retain inorganic anions, such as Cl^−^ and SO_4_^2-^ in the samples. The material with octadecyl (non-end-capped) functionalised trifunctional silane was activated by ionization of polar functional groups in the process.

The best operating material in terms of enrichment of the homologous carboxylates, repetition, and low matrix effects was Oasis HLB. The SPE optimization was made with 5 mg/L standards for 6 times. The average values of recovery RSD% in the SPE analysed with CE were 87, 9.0, 4.0, 5.9, 10.0, 6.0, 5.8, 8.1, and 19.7 for C_3_ to C_10_, respectively. Standard deviation on the average was 1-2%. Because the correlation was linear as a function of concentration and the 5 mg/L concentration (6.8 nL into separation) was low enough to show the working at low concentration range for the aerosol samples, the methods development was accepted with Oasis HLB material.

With the fast CE analysis the inorganic anions complicated the identification of C_2_ and C_3_. However, by using of SPE methods C_2_ and C_3_ could also be quantified, although the SPE technique did not improve their amount from that obtained with US. Therefore, the recoveries of DCAs were remained on the same level obtained with the US method. However, in SPE enrichment the concentrations of C_4_–C_10_ were remarkably improved.

**Figure 5:**
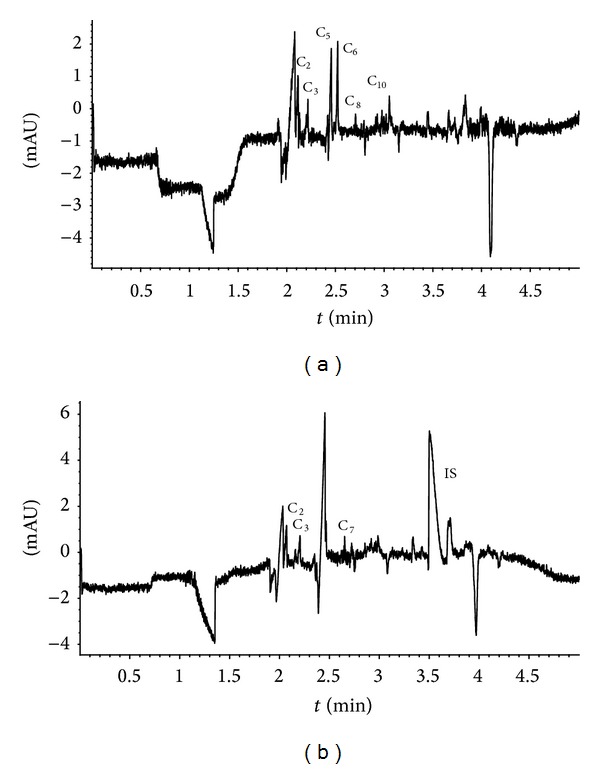
Electropherograms of (a) the air sample 2 and (b) the air sample 3 after solid-phase extraction. Sample collected with a cascade impactor (12 stages). The analyses were performed with two replicates and six repetitions. Electrolyte: 4 mM PDA + 0.5 mM MTAH, pH 11.0; indirect detection at 310/266 nm; sample injection for 6 sec at 50 mbar; separation voltage −24 kV; analysis time 5 min. Analysis conditions as described in Experimental.

The SPE eluent volume was 5000 *μ*L and that of the effluent less. To obtain quantitative results it was evaporated totally under nitrogen and the residue was dissolved into 50 *μ*L (minimum volume of sample in our optimised CE method) of Milli-Q water to enrich the acids. The results were calculated from three samples (50 *μ*L) treated with every Oasis extraction. The efficiencies with SPE clean-up were mainly between 26 and 134% for the DCAs. Depending on the concentration of acid anions in the aerosol samples, in CE analysis inorganic compounds were not always separated from the first two migrating DCAs (C_2_ and C_3_), which is why the BGE needed further optimisation (Figure 5). Finally, the repeatability of the analyses was acceptable. The calibration curves and other validation parameters for the studied compounds under the final conditions are presented in Table 1.

### 3.5. Aerosol Sample Determination

After profound optimisation of the capillary electrophoresis method, sensitivities of the DCAs were improved with 50% for the DCAs compared to our earlier results [8]. The reason was the using of another salt of the surfactant in the separation method: instead of MTAB, low sensitive MTAH was used. In addition, due to sample processing, inorganic sulphate and chloride of the aerosol in aqueous media could be removed from the final samples by extracting with SPE.

Our results agreed that the amounts of DCAs are very small in the outdoor air samples. As can be seen from Figures 5(a) and 5(b) the performance of the capillary electrophoresis was good. Furthermore, a great improvement in the sensitivity could be obtained via the sample preparation procedure that maximizes the DCAs signals (~100 times). In spite of the improvements, only some of the DCAs were determined from the aerosol samples. Because of the indirect UV detection the DCAs in the air samples were identified with spiking of their mixture at 5 mg/L concentration. The advantage of the sample clean-up and enrichment was the exhaustion of the inorganic species from the sample to obtain electropherogram baseline without great variation.

The CE data showed that the main compounds in the samples were malonic (C_3_) and glutaric (C_5_) acids, which were found in the water-soluble fraction of the aerosol samples. However, also minor amounts of some other CDAs could be quantified, namely, C_2_, C_6_–C_8_, and C_10_ (Table 2). The concentrations were calculated to be 5–10 ng/m^2^ (Table 3), when DCAs were detected from the isolated and SPE (Oasis) treated aerosol samples and the procedure to enrich the samples was used. The detection limits (signal-to-noise ratio 3) were at pg level, the highest malonic acid (C_3_) and the lowest suberic acid (C_8_) (Table 1).

## 4. Conclusions

A simple and repeatable isolation-separation-concentration combination was developed for the determination of homologous dicarboxylic acids from aerosol samples. Overall, the methodology provides efficiency and sensitivity to the separation and provides fast analysis times with repeatability in high alkaline solutions and economical analysis of DCAs from aerosol particles. Furthermore, after the concentration of the samples the method is relatively sensitive, when the DCA group specific CE method is used. The pretreatment methods used are supposed to be online coupled with online CE instruments in the future. When the technology is working, the reproducibility of the separation is supposed to be good enough for monitoring the environmental aerosols better and with lower costs than now.

## Figures and Tables

**Table 1:** Method optimization data: the calibration of the DCAs. Experimental conditions: 4 mM PDA + 0.5 mM MTAH, pH 11.0; indirect detection at 310/266 nm (pilot/reference wavelength); sample injection for 6 sec. at 50 mbar; separation voltage −24 kV; analysis time 5 min.

Dicarboxylic acids	Concentration calibration equation	*R* ^2^	Calibration range* [pg]	Minimum amounts (MDL)** [pg]
Oxalic acid (C_2_)	*y* = 0.2038*x* + 0.335	0.9996	1.03*–102.6	18.3**
Malonic acid (C_3_)	*y* = 0.1509*x* + 2.8785	0.9337	1.19–118.9	20.1
Succinic acid (C_4_)	*y* = 0.3199*x* − 0.0787	0.9994	1.02–102.6	15.2
Glutaric acid (C_5_)	*y* = 0.1107*x* + 1.4553	0.9367	1.02–101.6	11.4
Adipic acid (C_6_)	*y* = 0.1995*x* + 0.2539	0.9989	1.01–100.8	11.9
Pimelic acid (C_7_)	*y* = 0.1995*x* + 0.2539	0.9989	1.02–102.0	12.2
Suberic acid (C_8_)	*y* = 0.2064*x* + 1.4553	0.9978	1.0–100.0	15.2
Azelaic acid (C_9_)	*y* = 0.179*x* + 0.3412	0.9991	1.07–107.3	17.4
Sebacic acid (C_10_)	*y* = 0.1855*x* + 0.0131	0.9993	1.03–103.0	30.5
Mandelic acid (MA, IS_1_)	*y* = 0.1282*x* + 0.1979	0.9908	nd	nd
Trichloroacetic acid (TCA, IS_2_)	*y* = 0.031*x* + 0.3666	0.9778	nd	nd

*Example about the calculations: Injection of 0.5 p.s.i. for 6 sec. 6 nL sample into the capillary, calculated with program CE Expert Lite; corresponds: 0.167 mg/L standard sample that is injected for 6 s at 0.5 p.s.i.; signal-to-noise ratio (S/N) used is 3. Oxalic acid in detection: 1.03 pg.

**Calculation made by similar ways as in calibration; signal-to-noise ratios (S/N) is 3; Oxalic acid in the filter sample: 3.05 mg/L.

**Table 2:** The intraday and interday results of the aerosol samples separated and determined under optimized capillary electrophoresis conditions (see Table 1).

Dicarboxylic acids	Intraday RSD% (*n* = 6)		Interday RSD% (*n* = 9)	
	Migration time *t*_*M*_	Peak area Area	Migration time *t*_M_	Peak area Area
Oxalic acid (C_2_)	0.18	3.56	0.38	3.11
Malonic acid (C_3_)	0.0	7.50	0.15	5.62
Succinic acid (C_4_)	0.03	8.95	0.31	6.63
Glutaric acid (C_5_)	0.04	3.99	0.42	2.78
Adipic acid (C_6_)	0.05	5.86	0.45	5.20
Pimelic acid (C_7_)	0.05	10.0	0.50	8.70
Suberic acid (C_8_)	0.05	5.89	0.53	4.67
Azelaic acid (C_9_)	0.04	5.76	0.51	4.93
Sebacic acid (C_10_)	0.08	8.06	0.43	7.36
Trichloroacetic acid (TCA, IS_2_)	0.16	19.7	0.76	17.5

**Table 3:** DCAs in the aerosol samples studied. Samples were pretreated following the optimized concept informed in the experimental. Separation conditions as in Table 1.

Atmospheric aerosols	C_2_(ng/m^3^)	C_3_ (ng/m^3^)	C_4_ (ng/m^3^)	C_5_ (ng/m^3^)	C_6_ (ng/m^3^)	C_7_ (ng/m^3^)	C_8_ (ng/m^3^)	C_9_ (ng/m^3^)	C_10_ (ng/m^3^)
Air sample 1 (1st lot)	2.64	<MDL*	1.85	2.73	nd	nd	<MDL****	nd	nd
Air sample 2 (2nd lot)	3.66	4.21	nd	nd	nd	1.71	nd	nd	1.94
Air sample 3 (3rd lot)	3.80	1.16	nd	4.35	6.25	nd	2.92	nd	1.85
Air sample 4 (4th lot)	<MDL**	<MDL*	nd	0.472	nd	<MDL***	nd	nd	0.477

*MDL 0.551 ng/m^3^; **MDL 0.477 ng/m^3^; ***MDL 0.472 ng/m^3^; ****MDL 0.463 ng/m^3^; signal-to-noise ratios (S/N) is 3.

^a^The samples were taken on different days.

^b^US + SPE means after ultrasonication of the sample it was treated with solid-phase extraction (Oasis).
